# Saliva as a Potential Diagnostic Tool to Evaluate Relationship between Oral Microbiome and Potentially Malignant Disorders for Prevention of Malignant Transformation

**DOI:** 10.31557/APJCP.2021.22.1.125

**Published:** 2021-01

**Authors:** Sandhya Singh Kushwaha, Rupinder Kaur Multani, Narendra Singh Kushwaha, Swati Gautam, Deepti Garg Jindal, Karandeep Singh Arora, Avijit Avasthi

**Affiliations:** 1 *Department of Oral Pathology and Microbiology, Faculty of Dental Sciences, King George Medical University, Lucknow, Uttar Pradesh, India. *; 2 *Department of Oral Pathology and Microbiology, Bhojia Dental College and Hospital, Baddi, Himachal Pradesh, India. *; 3 *Department of Prosthodontics, Bhojia Dental College and Hospital, Baddi, Himachal Pradesh, India. *; 4 *Department of Oral Medicine and Radiology, Bhojia Dental College and Hospital, Baddi, Himachal Pradesh, India. *; 5 *Department of Public Health Dentistry, Bhojia Dental College and Hospital, Baddi, Himachal Pradesh, India. *

**Keywords:** Oral microbiome, PMDs, OSCC, tobacco, saliva

## Abstract

**Objective:**

The current research was conducted with an aim to assess the association of oral microbiome with Potentially malignant disorders (PMDs) because usage of tobacco in any form alters the normal microbiome and shifts it towards dysbiosis. Thus, our definitive knowledge of the oral commensal bacteria and oral cancer link can definitely be used as a potential adjunct to early diagnosis and management of PMDs and prevent it’s malignant transformation.

**Study Design:**

A total of 100 individuals of minimum 18 years of age were included in the study which, were classified into 2 groups of tobacco users (50) and non-tobacco users (50). The tobacco users had a history of tobacco consumption for at least 5 years.

**Results:**

The present study, showed highest percentage (72%) of anaerobic bacteria, followed by aerobic (22%) and lowest count of yeast (4%).

**Conclusion:**

The ecological shift to dysbiosis is a significant finding in oral carcinogenesis. Further investigation on a larger group of altered microbiomes will definitely help in establishing relationship of altered microbiome and PMDs, which can help in appropriate treatment and better prognosis.

## Introduction

A diverse and peculiar habitat is provided by the heterogenous nature of the oral cavity to the microorganisms present in the mouth with a vast distribution (Gaonkar et al., 2018). Microorganism persistent in the oral cavity are referred by a variety of names including oral microflora, microbiota or microbiome. Joshua Lederberg coined the term microbiome which, signifies the ecological community of commensal, symbiotic and pathogenic microorganisms coexisting in a human (Dewhirst et al., 2010). Risk of oral cancer is not only increased by the presence of microbiota in the oral cavity when activated by alcohol and smoking related carcinogens but, in addition, it is intricated with metastatic cancers which, hints towards the involvement of systemic mechanisms with carcinogenesis originating from oral microbiome (Gaonkar et al., 2018). 

A disturbance in the equilibrium of typical microbiome also called as dysbiosis is supposed to be linked with the disease. The gripping fact here is that, with time, oral microbiomes has a tendency to manifest intra-individual stability which, advocates the fact that disruption of microbiome can serve as potent identifier for diseased condition (NLM, 2020). 

A vast variety of bacteria is present in the mouth of a healthy person mostly commensals which, maintain the homeostasis including protecting against pathogens; down-regulation of inflammation and cytokine production; and decreasing levels of nitrate and conversion of nitrite to nitrogen oxide and other reactive intermediates (Hernandez et al., 2017). 

The most common condition pertinent to the oral cavity in terms of potentially malignant disorders (PMDs) include leukoplakia, OSMF, tobacco pouch keratosis, and erythroplakia but, it is also a fact that almost 50% of the oral squamous cell carcinomas (OSCCs) originate from normal mucosa. The prognostic significance of an individual lesion is difficult to determine (NLM, 2020). Malignant transformation rate of oral leukoplakia ranges from 0.13 to 17.5% and that of OSMF varies from 4.5% -7.6%.

In case of predecessor typically known as oral epithelial dysplasia (OED), malignancy can be predicted by a slightest change in the composition of oral microbiota (NLM, 2020). 

Saliva has been considered as a diagnostic tool for enormous conditions. Here, saliva remains directly in contact with the OSCC and the precursor lesions, which makes easy detection of DNA, RNA and proteins released by these lesions. Moreover, detection of these agents from saliva can be done at an early stage and this technique can put an end to the expensive and invasive procedure such as biopsies and on top of it prognosis of the lesion can be improved (Lee et al., 2017). 

Hence, keeping in mind the fact that initial screening and assessment of PMDs can be vital in preventing their malignant conversion into OSCC the current research was conducted with an aim to assess the association of oral microbiome with Potentially malignant disorders (PMDs) because usage of tobacco in any form alters the normal microbiome and shifts it towards dysbiosis.

## Materials and Methods

The study was conducted after approval from Bhojia Dental College and Hospital Ethical Committee vide letter no. BDC/BUDH/1181 dated 21.02.2019. A total of 100 individuals were taken up in the study and divided into 2 groups, where group 1 consisted of tobacco users (50) and group 2 consisted of non-tobacco users (50). 

Individual aged 18 years and above who had been using tobacco (in any form) regularly for at least 5 years were included in the study. Patients receiving antimicrobial therapy, radiotherapy, chemo/immunotherapy, patients with systemic condition effecting immune system like diabetes, patients with mucosal lesions and patients on immunosuppressive or antibiotic drugs were excluded from the study. In addition to the exclusion criteria female patients were also excluded because in terms of tobacco consumption they represent a small amount and thus making it difficult to match. 

Unstimulated saliva collection was done using a sterilized swab. Then, immediately, the saliva soaked swab was passed to Robertson’s cooked meat media and incubated at a temperature of 37°C for next 24 hours. After completion of incubation, turbidity is evident in the medium, which was further inoculated on blood agar and MacConkey agar plates using the sterile inoculating loop which holds 0.002 ml. Blood agar plates were incubated in CO_2 _jar at 37°C for 18-24 hours. These agar plate were further incubated overnight at a temperature of 37°C. Once incubation colony characteristics became evident, Gram staining was carried out. The identification of organisms was done using standard biochemical tests When culture of material containing growth was carried out using plating, growth of different bacteria formed separate colonies, each of which was usually a pure culture descended from a single inoculated cell. Routinely, isolation was carried out sub-culture from the plate having well differentiated colonies. A solitary colony suspected to be of pathogenic organism based on its appearance, was subjected to sub-cultured in a tube or a plate of sterile culture medium. Isolates were identified macroscopically, microscopically and biochemically. Isolates were examined macroscopically with unaided eye for colour, size, consistency, shapes, odour and microscopically by Gram staining technique and Lactophenol cotton blue mounts. For biochemical analysis tests were carried out which included Coagulase test, Catalase test, Citrate utilization, Indole test, Oxidase test, Urease test, inoculation on Kligler Iron Agar (KIA) and germ tube test for Candida species.

The readings were recoded and tabulated using MS Excel (Microsoft Office 2010, Washington, USA) and the readings were subject to statistical analysis using Chi-square test which, was used to test significance comparison between percentage of groups. The statistical analysis was performed using SPSS software (v19; SPSS, Chicago, IL, USA). 

## Results

**Figure 1:**
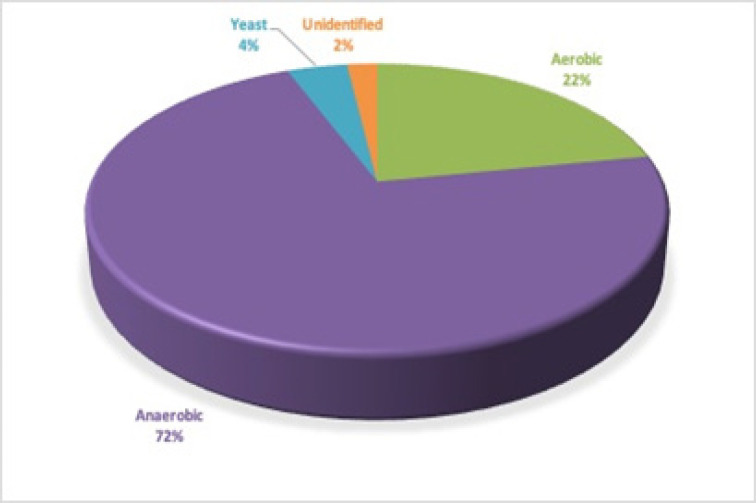
1 Distribution of Aerobic, Anaerobic Bacteria & Yeast in Oral Samples of All Subjects

Anaerobic bacteria showed highest percentage (72%) followed by aerobic bacteria (22%) and lowest in yeast (4%) (p<0.05) and the remaining 2% were unidentified (Figure 1). 

**Figure 2:**
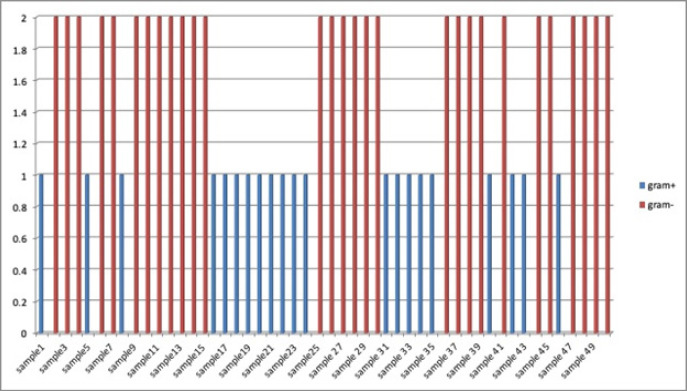
Distribution of Gram-Positive & Gram-Negative Bacteria in Group 1

**Figure 3:**
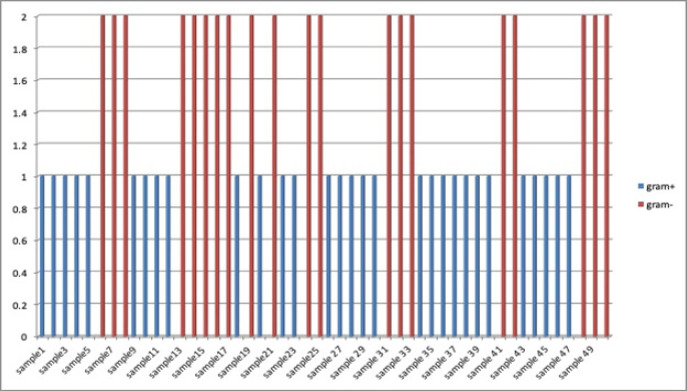
Distribution of Gram Positive & Gram Negative Bacteria in Group 2

Among group 1 participants more percentage of gram negative bacteria was observed as compared to gram positive, whereas reverse was true in case of group 2 users (Figures 2 and 3).

**Figure 4:**
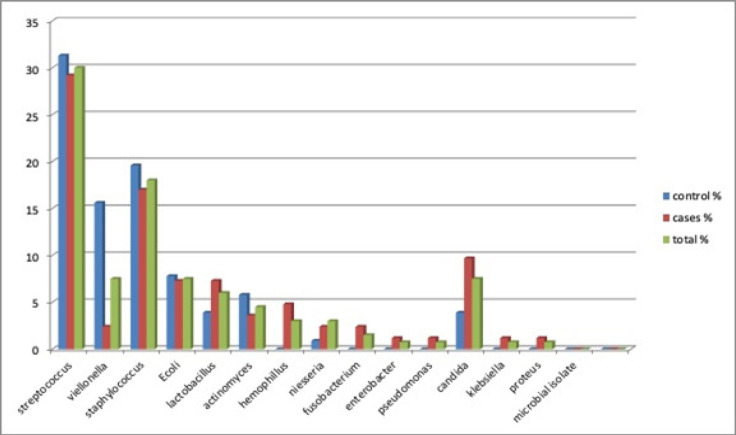
Distribution of Bacterial & Candida Isolates from Oral Samples of Both Group 1 and Group 2

The highest bacterial load consisted of normal oral flora whereas an unexpected spectrum of pathogenic and opportunistic microorganisms was found in the oral cavity of tobacco users including gram-positive and negative bacteria as well as yeasts. 31.3% of *Streptococcus* spp. was predominant in non tobacco users than tobacco users (29.2%) (Figure 4). Tobacco users had a higher percentage of aerobic bacteria compared to non tobacco users where as anaerobic bacteria had a higher percentage in non-tobacco users compared to tobacco users. There was a statistically significant association between oral potentially malignant disorders due to tobacco usage and microbial isolates. 

## Discussion

This study was undertaken to gain a clear picture of the microbial populations that may be present in potentially malignant disorders (PMDs) like oral leukoplakia, OSMF, tobacco pouch keratosis, erythroplakia, etc using tobacco products in any form i.e. smoking or smoke-less.

In this study higher rates of microbes (61.6%) were recovered from the oral cavity of subjects consuming tobacco products than control subjects (38.3%). This finding is in accordance with Kubota et al., (2011); Ogba et al., (2017); Saleh et al., (2016), who found that the incidence rate of bacteria were higher in tobacco users. However, these results are in contrast to the results of Sreedevi et al., (2012) who reported no difference in the occurrence of microbiota between tobacco users and non tobacco users. This difference may have resulted due to the types of sample obtained for analysis (Ogba et al., 2017).

In this study, *Hemophilus* species (4.9%), *Neisseria* species (2.4%), *Pseudomonas* species (1.2%), *Klebsiella* species (1.2%), *Candida* species (9.7%) were the most prevalent bacteria among tobacco users while *Streptococcus* species (31.3%), *Staphylococcus* species (19.6%), *Veillonella* species (15.6%) were found to be higher in non-tobacco users. Our findings somewhat differ from the reports of Wetzel et al., (2005) who isolated *Streptococcus* species, *Staphylococcus* species, *Pseudomonas* species as the most prevalent among tobacco users. This slight variation of microbial isolates may be because of variation in oral hygiene habits. 

Tobacco use, including cigarette smoking and tobacco chewing, is the primary cause of oral cancer worldwide (Humans, 2012). A significant change has been observed in the oral microbiome associated with smoking tobacco with significant variations in the abundance of common taxa. The fact may be true that a decrease in commensal species makes way for existence of harmful bacteria, although there was no elevation of any particular pathogenic species in the present study (Wu et al., 2016). Contrastingly, changes in the normal microflora of the oral cavity might alter its ability to act against the inflammation associated with betel nut resulting in higher rate of malignant transformation (Hernandez et al., 2017).

A “healthy” oral microbiome is balanced, stable, and in a symbiotic state, however, the presence of certain factors, such as poor diet, illness, and stress, disrupts the balance and shifts the oral microbiome to a state of dysbiosis and results in shifted diversity and proportions of microorganisms found in the oral cavity (Coates et al., 2017). 

An early detection not only reduces the severity and complications of the lesion but, also, is an important factor for success of the therapy. Over the last few decades the potency of saliva as a diagnostic tool has been studied extensively considering the large number of advantages such as easy sampling, collection procedure is non-invasive, inexpensive, real time diagnostic values and so on. Moreover, there is presence of vast variety of biomarkers including genetic and protein materials (Javaid et al. 2015).

In addition, saliva has been considered as a source of nutrition for the oral microbiome along with antimicrobial factors which, is importance for the maintenance of microbial homeostasis (Lira-junior et al., 2018).

A lot of factors have been considered as contributing factor associated with altered salivary bacterial profiles including periodontitis and caries. Moreover, other factors have also been associated with change in the oral microbiome which includes smoking and some of the underlying systemic conditions like inflammatory bowel disease. However, till date the specificity has not been taken into consideration and not properly addressed. Other factors that are considered to shape the oral microbiome may include environmental factors pertaining to a person’s systemic health, genetic predisposition, and dietary habits. On continuous research, it has been evident that the physiology and immunity of a person is dependent on the oral microbiome, which, in turn highlights the importance of evaluating the salivary microbiota profiles in different settings (Lira-junior et al., 2018).

In conclusion, thus, the present study successfully demonstrates that there is a relationship between oral microbiota and potentially malignant disorders using saliva with the increase or decrease in species of microorganisms. The ecological shift to dysbiosis is a significant finding in oral carcinogenesis. However, further, detailed study of the altered oral microbiota by isolating the various strains associated with PMDs and OSCC will be beneficial in the assessment of their definite role in carcinogenesis. Thus, variations in the normal oral microbiota can serve as a potential diagnostic indicator for PMDs. 
